# Patients with more comorbidities have better detection of chronic conditions, but poorer management and control: findings from six middle-income countries

**DOI:** 10.1186/s12889-019-8112-3

**Published:** 2020-01-06

**Authors:** Grace Sum, Gerald Choon-Huat Koh, Stewart W. Mercer, Lim Yee Wei, Azeem Majeed, Brian Oldenburg, John Tayu Lee

**Affiliations:** 1 Saw Swee Hock School of Public Health, National University of Singapore, 12 Science Drive 2, Tahir Foundation Building, Singapore, 117549 Singapore; 2 Primary Care and Multimorbidity, Usher Institute of Population Health Sciences and Informatics, University of Edinburgh, Edinburgh, Scotland; 3 Yong Loo Lin School of Medicine, National University of Singapore, Singapore, Singapore; 4 Department of Primary Care and Public Health, School of Public Health, Imperial College London, London, England; 5 Nossal Institute for Global Health, Melbourne School of Population and Global Health, University of Melbourne, Melbourne, Australia

**Keywords:** Comorbidity, Non-communicable diseases, Chronic conditions, Ageing, Health monitoring, Access to care

## Abstract

**Background:**

The burden of non-communicable diseases (NCDs) is rising rapidly in middle-income countries (MICs), where NCDs are often undiagnosed, untreated and uncontrolled. How comorbidity impacts diagnosis, treatment, and control of NCDs is an emerging area of research inquiry and have important clinical implications as highlighted in the recent National Institute for Health and Care Excellence guidelines for treating patients suffering from multiple NCDs. This is the first study to examine the association between increasing numbers of comorbidities with being undiagnosed, untreated, and uncontrolled for NCDs, in 6 large MICs.

**Methods:**

Cross-sectional analysis of the World Health Organisation Study of Global Ageing and Adult Health (WHO SAGE) Wave 1 (2007–10), which consisted of adults aged ≥18 years from 6 populous MICs, including China, Ghana, India, Mexico, Russia and South Africa (overall *n* = 41, 557).

**Results:**

A higher number of comorbidities was associated with better odds of diagnosis for hypertension, angina, and arthritis, and higher odds of having treatment for hypertension and angina. However, more comorbidities were associated with increased odds of uncontrolled hypertension, angina, arthritis, and asthma. Comorbidity with concordant conditions was associated with improved diagnosis and treatment of hypertension and angina.

**Conclusion:**

Patients with more comorbidities have better diagnosis of chronic conditions, but this does not translate into better management and control of these conditions. Patients with multiple NCDs are high users of health services and are at an increased risk of adverse health outcomes. Hence, improving their access to care is a priority for healthcare systems.

## What we know now and knowledge gaps

- The burden of non-communicable diseases (NCDs) is rising rapidly in middle-income countries (MICs), where NCDs are often undiagnosed, untreated and uncontrolled.
- From the limited number of studies in high-income countries, there is preliminary evidence that more comorbidities negatively impact treatment and control of NCDs. There are also mixed results on how concordant and discordant comorbidities influence treatment and control.
- There is a specific knowledge gap in MICs on how more comorbidities with a NCD is associated with the diagnosis, treatment, and control of the NCD.
- There is also a knowledge gap in MICs on how concordant and discordant comorbidities with a NCD are associated with the diagnosis, treatment, and control of the NCD.

## What this study adds

- A higher number of comorbidities was associated with better diagnosis of some NCDs.
- However, this did not translate into better management and control of NCDs. More comorbidities associated with even worse control of NCDs.
- Comorbidity with concordant conditions was associated with improved diagnosis and treatment, compared to comorbidity with discordant conditions.
- Patients with more comorbidities have better diagnosis of chronic conditions, but this does not translate into better management and control of these conditions. Patients with multiple NCDs are high users of health services and are at an increased risk of adverse health outcomes. Hence, improving their access to care is a priority for healthcare systems.

## Background

Non-communicable diseases (NCDs) are the leading cause of global disease burden with 85% of premature mortality due to NCDs occurring in low- and-middle income countries [1]. In middle-income countries (MICs), there is a high prevalence of multiple chronic conditions in young adults, and not only in the elderly [2]. Poor chronic disease outcomes in MICs pose a major hurdle to attain the health target 3.4 of United Nation’s Sustainable Development Goals, which is to reduce premature mortality from NCDs by a third by 2030 [3].

How comorbidity, defined as the presence of co-occurring NCDs in an individual [4, 5], impacts diagnosis, treatment, and control of NCDs is an emerging area of research inquiry and has important clinical implications as highlighted in the recent National Institute for Health and Care Excellence guidelines for treating patients suffering from multiple NCDs [6]. A small number of studies in high-income countries show mixed results on the implications of comorbidities on the management and control of single NCDs, such as hypertension or diabetes [7–10]. A study in the United States revealed that the proportion of persons with uncontrolled hypertension increased as the number of unrelated comorbid NCDs increased [9].

In contrast to this finding, a recent ecological study in the United Kingdom found that unrelated and related co-occurring NCDs could either be associated with better or worse treatment outcomes for patients with diabetes [8]. To the best of our knowledge, there are very few studies on this topic in MICs, and there is a need to fill the gap on comorbidities in MICs [11]. Findings from the small number of studies in high-income countries may not be applicable to MICs, as their health systems vary substantially and patients in MICs tend to have less frequent visits with well-trained healthcare providers [2, 12].

This study aims to examine how more comorbidities is associated with being undiagnosed, untreated, and uncontrolled for NCDs in 6 high-population MICs. We also investigate the odds of NCDs being undiagnosed, untreated, and uncontrolled, when comorbidities are concordant versus discordant. This paper considers the implications of the research findings for clinicians and policymakers on the revision of health structures and policies to improve management and control of NCDs in the context of MICs.

## Methods

### Sample and data

We used cross-sectional data from the World Health Organisation Study of Global Ageing and Adult Health (WHO SAGE) Wave 1 (2007–10) which collected nationally representative samples of people aged 50+ years in China, Ghana, India, Mexico, Russia and South Africa, with a smaller sample of adults aged 18–49 years in each country for comparison [13]. SAGE contains information on sociodemographic characteristics, anthropometrics and biomarkers, NCDs, healthcare utilisation, quality of life and well-being, social cohesion, and impact on caregiving [13]. Face-to-face interviews were conducted in all countries, using a combination of computer-assisted personal interview, and paper and pencil [13].

The original total sample size of adults aged ≥18 years was 44,089 (China: 15,009, India: 12,198, Ghana: 5563, Russia: 4350, Mexico: 2744, South Africa: 4225). We excluded those who had missing values on outcome variables and covariates (5.7% of entire sample). Final sample size was 41,557 (China: 14,906, India: 11,159, Ghana: 5067, Russia: 4330, Mexico: 2618, South Africa: 3477).

### Variables

Figure 4 in Appendix summarises predicting variables and outcomes.

#### Chronic conditions

SAGE collected information on 9 NCDs including hypertension, angina, arthritis, asthma, chronic lung disease (CLD), diabetes, cataract, stroke, and depression. All 9 NCDs had questions on self-reported diagnosis and treatment. Subjects self-reported the NCD if they answered affirmatively to: “Have you ever been diagnosed with …? ”. Subjects self-reported being treated for the NCD if they answered affirmatively to: “Have you been taking medication or other treatment for it during the ( … last 2 weeks / … last 12 months)?”

SAGE had symptom-based assessment or physical measurements for only 6 of 9 NCDs. Hence only these 6 of 9 NCDs could be assessed in this study for being undiagnosed, or for being uncontrolled. These were hypertension, angina, arthritis, asthma, CLD, and depression.

For hypertension, physical measurement of blood pressure was taken, and subjects with hypertension had systolic blood pressure ≥ 140 mmHg or diastolic blood pressure ≥ 90 mmHg [14]. For angina, depression, arthritis, asthma, and CLD, symptom-based assessments were according to validated symptom scales derived through a standard algorithm based on a set of symptomatic questions from SAGE survey (i.e. Rose questionnaire for angina [15, 16], Composite International Diagnostic Interview for depression [17, 18], receiver operating characteristic curve analysis that generated an algorithm for arthritis diagnosis by symptoms [19]). These methods were consistent with SAGE individual country reports published by WHO [20, 21]. Table 3 in Appendix shows details on the symptom-based assessment.

#### Predicting variables

The first predicting variable was number of comorbidities. Subjects were categorised by number of diagnosed comorbidities: 0, 1, 2, 3 and 4+ comorbidities. The second predicting variable was co-occurrence of each NCD with only concordant NCDs, only discordant NCDs, and only depression. Concordant NCDs were those that represented parts of the same overall pathophysiologic risk profile [7]. For example, studies considered diabetes and hypertension “concordant” due to similar pathophysiologic risk profiles or were more likely the focus of a similar disease complex and management plan [7, 22]. Conditions considered “discordant” were not directly related in pathogenesis or did not share an underlying predisposing factor [9]. For example, asthma and arthritis are considered “discordant” to diabetes and hypertension [9]. For our study, concordant sets of NCDs included (i) hypertension, angina, stroke, diabetes [9, 22]; (ii) diabetes, cataract [23]; (iii) asthma, CLD [24]. Two NCDs, arthritis and depression, did not have any concordant NCDs.

#### Outcomes

Firstly, we examined the associations between being undiagnosed, untreated, and uncontrolled for NCDs with increasing comorbidity, and secondly, the associations between being undiagnosed, untreated and uncontrolled for NCDs when the NCD co-occurs with only concordant NCDs, only discordant NCDs, and only depression.

Undiagnosed subjects did not have self-reported diagnosis of the NCD by a medical professional but had the NCD based on SAGE assessment. Untreated subjects self-reported previous diagnosis of the NCD by a medical professional but self-reported not having treatment (medications, lifestyle changes, therapy, and/or counselling). Subjects who did not have treatment in the last 2 weeks and in the last 12 months were referred to in this study as (‘unT-last 2 weeks’) and (‘unT-last 12 months’), respectively. Uncontrolled subjects self-reported being both diagnosed and treated for the NCD, but had symptoms of the NCD based on SAGE assessment. Uncontrolled subjects who had treatment in the last 2 weeks were referred to as ‘unC-T-last 2 weeks’ and those who had treatment in the last 12 months were referred to as ‘unC-T-last 12 months’. Figure 5 and Table 4 in Appendix describe the detailed definitions of being undiagnosed, untreated, and uncontrolled.

#### Covariates

Covariates were age (18-49 years, 50-64 years, 65 + years), sex, marital status (married, not married), education (primary or less, secondary, tertiary and above), wealth quintiles, residence (rural, urban), and health insurance (with/without insurance).

### Statistical analysis

We summarised subject characteristics by country with pooled data. For each NCD, we examined the prevalence of subjects with 0, 1, 2, 3, and 4+ comorbid conditions.

For the 6 NCDs that had symptom-based assessment, we presented the prevalence of undiagnosed subjects as their number of comorbidities increased from 0 to 1, 2, 3, and 4+. For all 9 NCDs, we presented prevalence of untreated subjects as the number of comorbidities increased from 0 to 1, 2, 3, and 4+. For the 6 NCDs that had symptom-based assessment, we presented the prevalence of uncontrolled subjects as the number of comorbidities increased from 0 to 1, 2, 3, and 4+. In addition, we presented the prevalence of being undiagnosed, untreated, and uncontrolled when each NCD co-occurred with only concordant NCDs, only discordant NCDs, and only depression.

We conducted a series of multivariable logistic regression analyses. For each NCD, we obtained adjusted odds ratios (AORs) of being undiagnosed, untreated and uncontrolled: Firstly, in association with greater number of comorbidities, and secondly, in association with the co-occurrence of only concordant NCDs, versus only discordant NCDs, and versus only depression. We adjusted for country fixed effects and covariates in all the regression models. The data analyses were weighted to account for the complex, multi-stage design of the SAGE survey. We performed statistical analyses using Stata 15∙1(StataCorp).

## Results

### Sample characteristics

We presented subjects’ characteristics by country in Table 1. Median age was 58 (IQR = 51–68) years. The prevalence of subjects with 2 or more NCDs was overall 19%, and was 38.7% in Russia, 17.7% in South Africa, 17.2% in Ghana, 16.8% in Mexico, 14.8% in India, and 13.9% in China. Using pooled data, 43.0% were male, 22.5% were aged above 70 years, 61.3% had primary school education or less, 18.8% were from the lowest income quintile, 52.5% resided in rural areas, and 48.1% did not have insurance. Table 5 in Appendix displays the prevalence of subjects with 0, 1, 2, 3, and 4+ comorbidities for each NCD.

**Table 1:** Sample characteristics of the population of China, India, Ghana, Russia, Mexico, and South Africa

	China	India	Ghana	Russia	Mexico	South Africa	Pooled
Total (N)	14,906	11,159	5,067	4,330	2,618	3,477	41,557
Sex (%)							
Male	46.7	38.74	52.65	35.64	38.20	39.72	43.01
Female	53.3	61.26	47.35	64.36	61.80	60.28	56.99
Marital Status (%)							
Not married	16.75	22.34	41.70	46.26	41.29	55.88	29.19
Married	83.25	77.66	58.30	53.74	58.71	44.12	70.81
Age Group (%)							
18-29	1.44	14.27	2.53	2.26	2.18	2.04	5.20
30-39	3.41	14.75	5.94	3.39	6.49	2.59	6.89
40-49	6.05	12.54	7.20	3.95	7.30	3.36	7.57
50-59	38.70	26.16	33.04	33.76	16.23	40.47	32.86
60-69	26.46	19.92	23.60	24.57	34.91	29.59	24.95
70+	23.94	12.37	27.69	32.06	32.89	21.94	22.53
Multimorbidity (%)							
0 NCDs	61.68	74.75	56.80	50.21	68.43	48.33	64.00
1 NCD	24.47	10.47	26.02	11.13	14.76	33.95	17.00
2 or more NCDs	13.85	14.78	17.18	38.66	16.81	17.72	19.00
Mean number of NCDs	1.03	1.41	1.23	1.88	1.36	1.40	1.11
Education Level (%)							
No schooling	23.93	45.18	50.74	0.95	17.07	24.04	30.09
Primary or lower	35.66	25.79	23.03	9.01	59.43	47.66	31.20
Secondary	21.27	12.47	5.51	18.15	10.62	14.75	15.44
Tertiary or higher	19.14	16.55	20.72	71.89	12.87	13.55	23.27
Wealth Quintile (%)							
Q1 (lowest)	19.03	17.85	19.44	17.78	20.59	20.13	18.82
Q2	19.82	19.23	19.62	19.40	20.55	20.19	19.67
Q3	20.01	19.14	19.76	19.98	18.56	19.64	19.62
Q4	20.66	21.02	20.70	20.35	20.66	20.07	20.68
Q5 (highest)	20.48	22.75	20.49	22.49	19.63	19.96	21.20
Location (%)							
Rural	50.87	74.53	59.05	24.32	26.70	33.62	52.49
Urban	49.13	25.47	40.95	75.68	73.30	66.38	47.51
Insurance (%)							
No insurance	12.97	95.91	63.77	0.48	No data	82.17	48.14
With insurance (mandatory/voluntary)	87.03	4.09	36.23	99.52	No data	17.83	51.86

*NCD* Non-communicable disease, *N* Sample size, *Q* Quintile

### Undiagnosed NCDs

Table 2 shows the prevalence of subjects undiagnosed for each NCD, which ranged from 42.8% for undiagnosed arthritis to 62.2% for undiagnosed angina.

**Table 2:** Prevalence of undiagnosed, untreated, and uncontrolled subjects for each non-communicable disease

		Non-communicable disease (%)								
		Hypertension (*n*= 9778)	Angina (*n*= 3274)	Arthritis (*n*= 540)	Asthma (*n*= 153)	Chronic Lung Disease (*n*= 2455)	Diabetes (*n*= 2735)	Cataract^a^ (*n*= 3739)	Depression (*n*= 1129)	Stroke (*n*= 1205)
Prevalence of subjects (%)	1. Undiagnosed	59.11	62.23	42.76	55.48	50.27	NA	NA	67.57	NA
	2a. unT-last 2 weeks (untreated in the last 2 weeks)	34.89	42.04	58.39	46.21	91.19	33.76	NA	70.45	57.66
	2b. unT-last 12 months (untreated in the last 12 months)	17.43	18.11	31.74	27.84	84.84	24.48	59.98	63.54	42.47
	3a. unC-T-last 2 weeks (treated in the last 2 weeks, and uncontrolled)	71.14	83.61	77.41	78.00	81.72	NA	NA	50.34	NA
	3b. unC-T-last 12 months (treated in the last 12 months, and uncontrolled)	66.82	77.77	72.81	77.89	70.05	NA	NA	62.21	NA

^a^Assessed untreated in the last 5 years

**Fig. 1:**
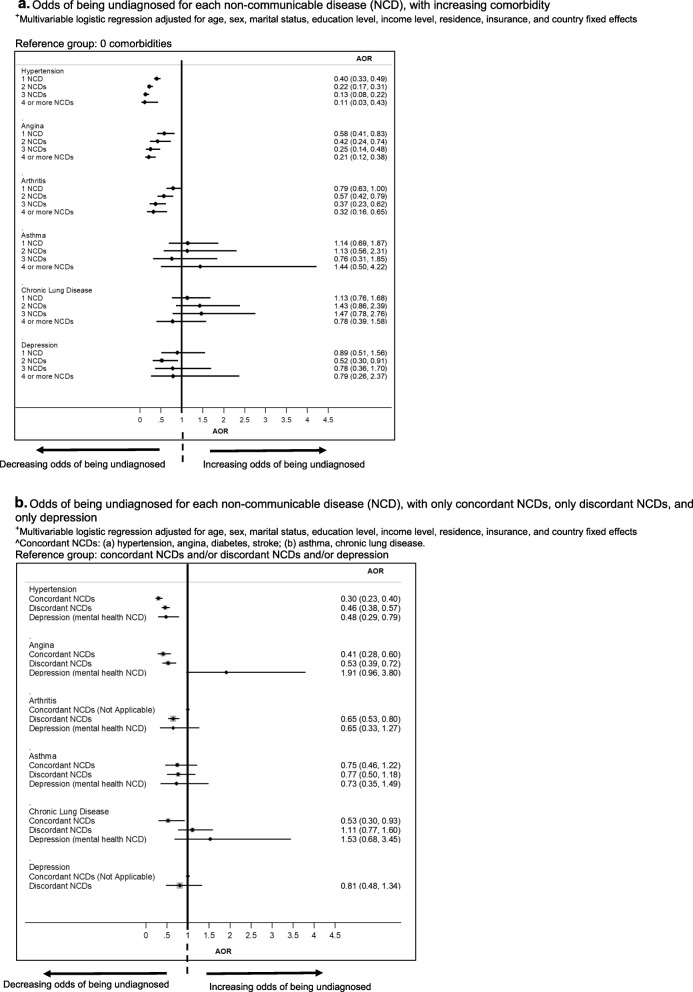
**a** Odds of being undiagnosed for each non-communicable disease (NCD), with increasing comorbidity. ^+^Multivariable logistic regression adjusted for age, sex, marital status, education level, income level, residence, insurance, and country fixed effects. **b** Odds of being undiagnosed for each non-communicable disease (NCD), with only concordant NCDs, only discordant NCDs, and only depression. ^+^Multivariable logistic regression adjusted for age, sex, marital status, education level, income level, residence, insurance, and country fixed effects. ^Concordant NCDs: (a) hypertension, angina, diabetes, stroke; (b) asthma, chronic lung disease

More comorbidities were associated with decreased odds of undiagnosed hypertension, angina and arthritis, but not for asthma, CLD, and depression (Fig. 1a). Comorbidity with concordant conditions was associated with decreased odds of undiagnosed hypertension and angina, but not for arthritis, asthma, CLD, and depression (Fig. 1b).

### Untreated NCDs

Table 2 shows the prevalence of subjects untreated for each NCD, with highest prevalence for CLD (91.2% ‘unT-last 2 weeks’, 84.8% ‘unT-last 12 months’), and lowest prevalence for diabetes (33.8% ‘unT-last 2 weeks’) and hypertension (17.4% ‘unT-last 12 months’).

**Fig. 2:**
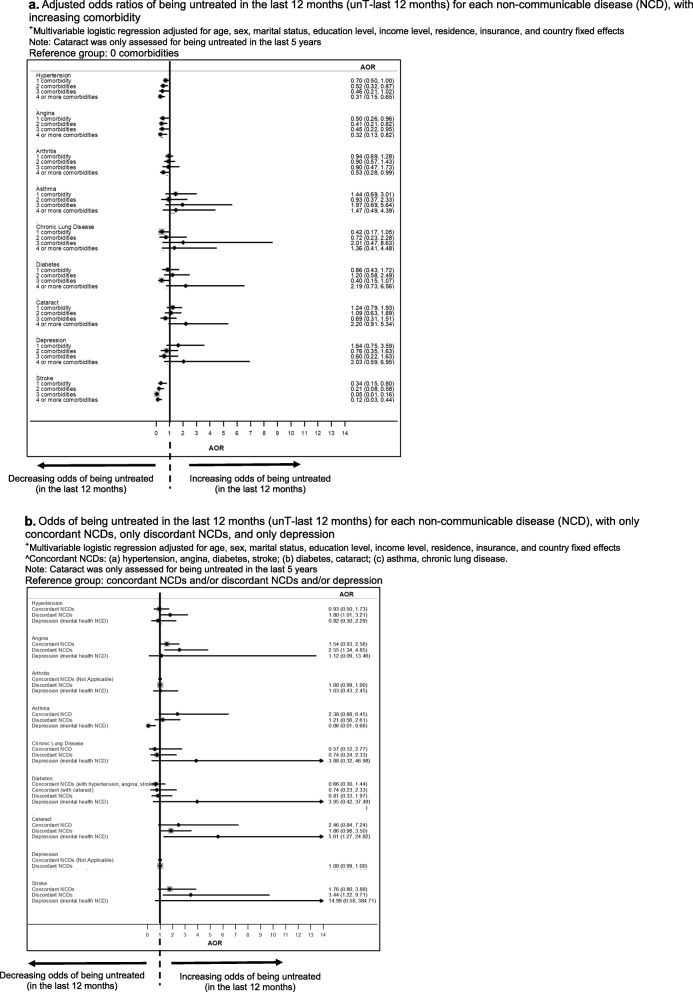
**a** Adjusted odds ratios of being untreated in the last 12 months (unT-last 12 months) for each non-communicable disease (NCD), with increasing comorbidity. ^+^Multivariable logistic regression adjusted for age, sex, marital status, education level, income level, residence, insurance, and country fixed effects. Note: Cataract was only assessed for being untreated in the last 5 years. **b** Odds of being untreated in the last 12 months (unT-last 12 months) for each non-communicable disease (NCD), with only concordant NCDs, only discordant NCDs, and only depression. ^+^Multivariable logistic regression adjusted for age, sex, marital status, education level, income level, residence, insurance, and country fixed effects^Concordant NCDs: (a) hypertension, angina, diabetes, stroke; (b) diabetes, cataract; (c) asthma, chronic lung disease. Note: Cataract was only assessed for being untreated in the last 5 years.

More comorbidities were associated with decreased odds of untreated hypertension and angina, but not arthritis, asthma, CLD, diabetes, depression and stroke for ‘unT-last 12 months’ (Fig. 2a). Similar results were seen for untreated hypertension and angina for ‘unT-last 2 weeks’ (Figure 6a in Appendix). In contrast, comorbidity was associated with increased odds of untreated diabetes.

Comorbidity with concordant conditions was associated with decreased odds of untreated hypertension and angina, but not arthritis, asthma, CLD, diabetes, depression and stroke (Fig. 2b, Figure 6b in Appendix).

### Uncontrolled NCDs

Table 2 shows the prevalence of subjects uncontrolled for each NCD, with highest prevalence for angina (83.6% ‘unC-T-last 2 weeks’; 77.8% ‘unC-T-last 12 months’), and lowest prevalence for depression (50.3% ‘unC-T-last 2 weeks’; 62.2% ‘unC-T-last 12 months’).

**Fig. 3:**
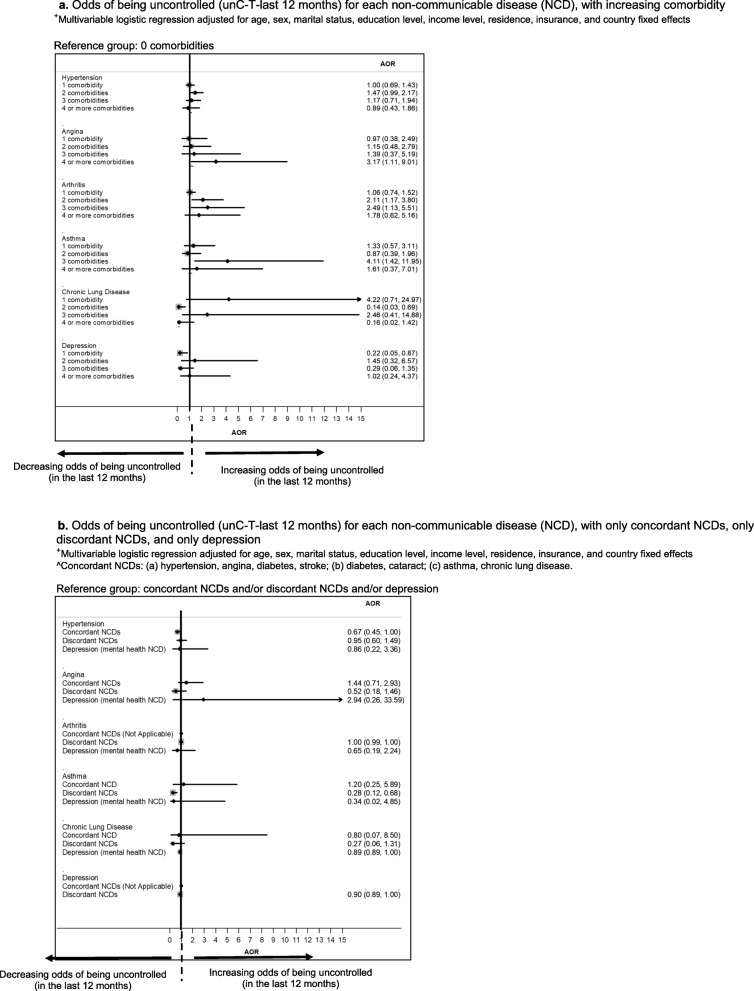
**a** Odds of being uncontrolled (unC-T-last 12 months) for each non-communicable disease (NCD), with increasing comorbidity. ^+^Multivariable logistic regression adjusted for age, sex, marital status, education level, income level, residence, insurance, and country fixed effects. **b** Odds of being uncontrolled (unC-T-last 12 months) for each non-communicable disease (NCD), with only concordant NCDs, only discordant NCDs, and only depression. ^+^Multivariable logistic regression adjusted for age, sex, marital status, education level, income level, residence, insurance, and country fixed effects. ^Concordant NCDs: (a) hypertension, angina, diabetes, stroke; (b) diabetes, cataract; (c) asthma, chronic lung disease

**Fig. 4:**
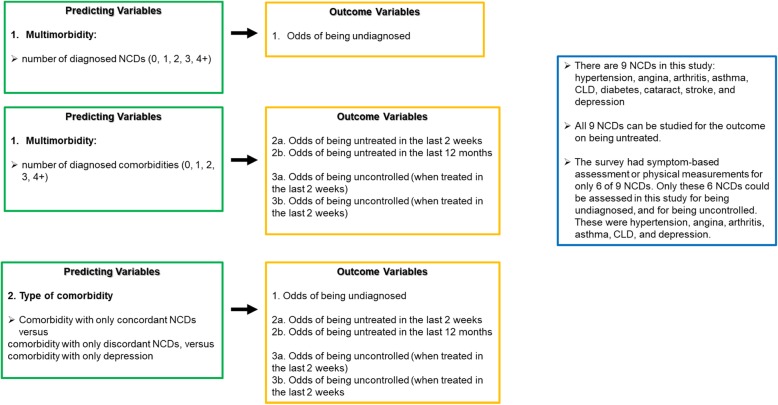
Predicting and outcome variables

**Fig. 5:**
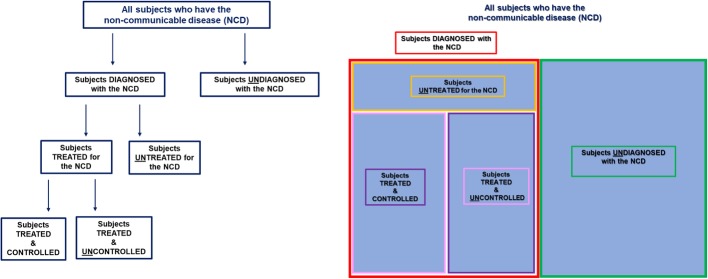
Classification of subjects who are undiagnosed, untreated, and uncontrolled

**Fig. 6:**
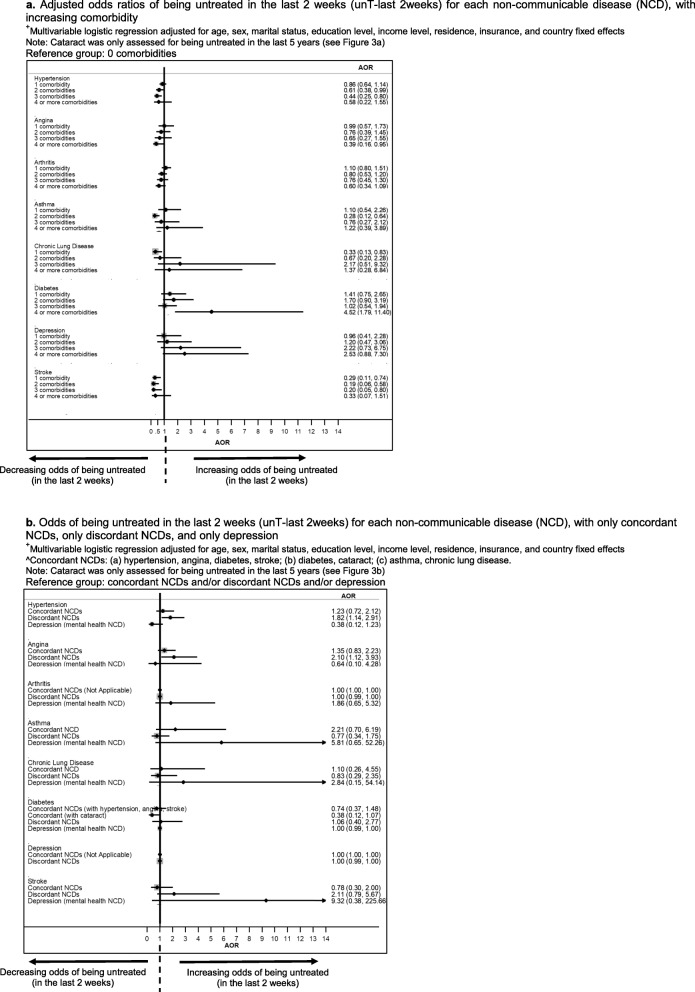
**a** Adjusted odds ratios of being untreated in the last 2 weeks (unT-last 2weeks) for each non-communicable disease (NCD), with increasing comorbidity. +Multivariable logistic regression adjusted for age, sex, marital status, education level, income level, residence, insurance, and country fixed effects. Note: Cataract was only assessed for being untreated in the last 5 years (see Figure 6a). **b** Odds of being untreated in the last 2 weeks (unT-last 2weeks) for each non-communicable disease (NCD), with only concordant NCDs, only discordant NCDs, and only depression. +Multivariable logistic regression adjusted for age, sex, marital status, education level, income level, residence, insurance, and country fixed effects. ^Concordant NCDs: (a) hypertension, angina, diabetes, stroke; (b) diabetes, cataract; (c) asthma, chronic lung disease. Note: Cataract was only assessed for being untreated in the last 5 years (see Figure 6b)

**Fig. 7:**
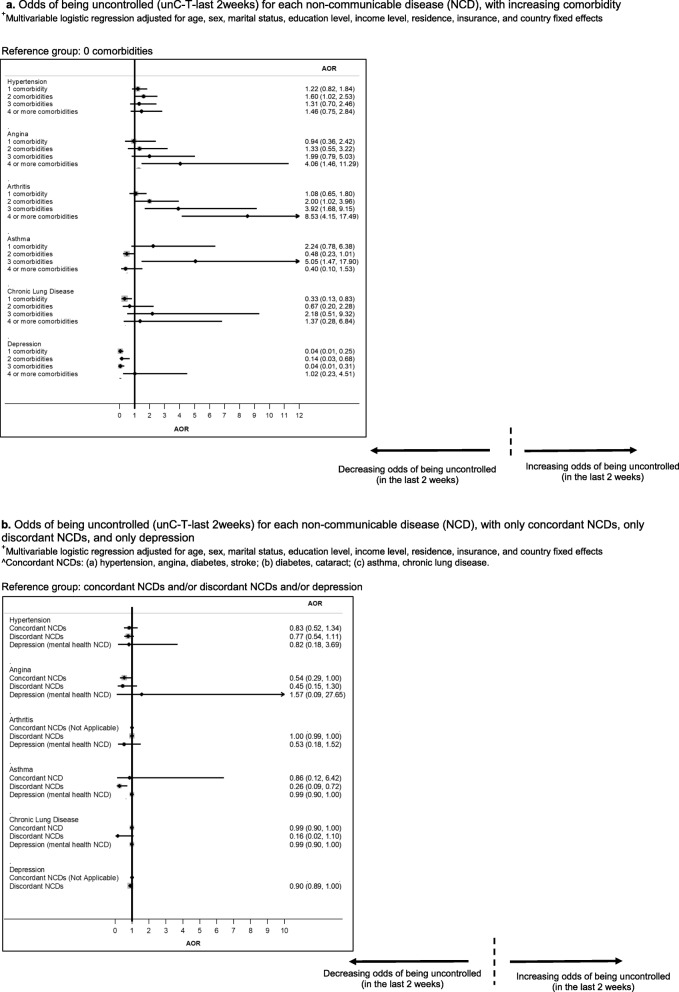
**a** Odds of being uncontrolled (unC-T-last 2weeks) for each non-communicable disease (NCD), with increasing comorbidity. +Multivariable logistic regression adjusted for age, sex, marital status, education level, income level, residence, insurance, and country fixed effects. **b** Odds of being uncontrolled (unC-T-last 2weeks) for each non-communicable disease (NCD), with only concordant NCDs, only discordant NCDs, and only depression. +Multivariable logistic regression adjusted for age, sex, marital status, education level, income level, residence, insurance, and country fixed effects. ^Concordant NCDs: (a) hypertension, angina, diabetes, stroke; (b) diabetes, cataract; (c) asthma, chronic lung disease

More comorbidities were associated with increased odds of uncontrolled hypertension, angina, arthritis, and asthma, but not CLD and depression for ‘unC-T-last 12 months’ (Fig. 3a). Similar results were seen for ‘unC-T-last 2 weeks’ (Figure 7a in Appendix).

Comorbidity with concordant conditions was not associated with decreased nor increased odds of being uncontrolled for all NCDs (Fig. 3b, Figure 7b in Appendix).

Tables 6-15 in Appendix show prevalence of being undiagnosed, untreated and uncontrolled for each NCD, in association with greater comorbidity, and with having only concordant NCDs, only discordant NCDs, and only depression.

## Discussion

### Principal findings

More comorbidities were associated with better diagnosis of hypertension, angina and arthritis, and better odds of having treatment for hypertension and angina. However, more comorbidities were associated with worse control of hypertension, angina, arthritis, and asthma.

Comorbidity with concordant conditions was associated with decreased odds of undiagnosed and untreated hypertension and angina. Comorbidity with concordant conditions was not associated with decreased nor increased odds of being uncontrolled for NCDs.

### Previous literature

Our finding on the positive effect of comorbidities on diagnosis is consistent with the small number of existing articles. Subjects with more comorbidities likely resulted in more frequent visits to, and interactions with multiple health providers. Healthcare professionals were more likely to detect previously undiagnosed co-occurring conditions [25, 26], such as hypertension, angina, and arthritis in this particular study. More comorbidities and increased frequency of healthcare visits were likely associated with a greater tendency for routinely taken blood pressure measurements to indicate hypertension, and for patients to self-report “chest pains” and arthritis symptoms [27–29]. For other conditions such as CLD and depression that require non-routine checks and are less self-reported, they may still be undiagnosed despite more patients having more comorbidities and more frequent healthcare visits [30–33].

Our finding on more comorbidities being associated with decreased odds of untreated hypertension and angina was not consistent with the small amount of existing literature, which mostly showed that comorbidity was associated with increased odds of untreated conditions. Existing studies showed that patients had difficulty coping with complex treatment regiments from polypharmacy, and had poor adherence from adverse drug events and high out-of-pocket expenditures on medicines [6, 34–36]. The difference in our findings and current literature is likely explained by our study examining only whether subjects were taking treatment or not, and did not assess if treatment was adequate, in terms of adherence to medicines (i.e. dose, frequency, duration, administration (e.g. techniques for subcutaneous insulin injections or inhaler use), route (oral, parenteral)), and to lifestyle modifications [37]. Our study showed that with more comorbidities, subjects had higher odds of taking treatment, but we expect that in reality, with more comorbidities, odds of treatment adherence and having adequate treatment would decline.

Our finding on more comorbidities being associated with poorer control is also consistent with the little amount of existing literature. The difficulty controlling NCDs tend to be exacerbated with more co-occurring conditions, and patients with comorbidities were less likely to have certain NCDs addressed [7, 9, 10]. Our study also showed that concordant comorbidities were associated with decreased odds of both undiagnosed and untreated hypertension and angina. Previously undiagnosed concordant NCDs may have a higher tendency to be simultaneously diagnosed during consultations with physicians for the primary NCD of interest, and concordant NCDs are more likely to have better co-ordination of care [6, 9, 38].

Our study’s finding that discordant conditions were not associated with poorer control of NCDs is generally in contrast to the literature. The small number of existing studies have revealed that discordant comorbidities, which have different pathophysiology and management plans, compromise the quality of care of the patient [7, 9, 39]. For example, a study in the United States on hypertension found that patients with discordant conditions were less likely to have controlled hypertension [9]. The existing literature is from high-income countries with likely higher adherence to treatment [40–42], and in contrast, adherence to treatment may be lower in MICs due to financial constraints and lower health literacy [36, 43]. Hence this non-adherence in MICs may explain the lack of difference between concordant versus discordant comorbidities in the association with control of NCDs.

### Strengths and limitations

This is the first study on high-population MICs that investigates the associations between comorbidity and the odds of being undiagnosed, untreated, and uncontrolled for NCDs. Self-reported diagnosis of NCDs may be under-reported and symptom-based assessment of NCDs may not correlate with true medical status [13, 44, 45]. Additionally, stigma could be a reason for under-reporting of depression in MICs [32, 46]. These limitations may have implications on under-estimations of associations in the study. However, previous work suggests this may not be a substantial problem as SAGE incorporated measures to minimise these issues [45]. There may be differential survey responses across countries. However, the survey methodology included strategies to detect and correct for systematic reporting biases in health interview surveys, such as vignette methods and objective performance tests [44]. Strategies were used to improve data comparability, such as utilising common definitions of concepts, common data collection methods and translations, rigorous sample design, and post-hoc harmonisation [44].

Additionally, this survey only asked if subjects were taking treatment (medicines, lifestyle changes), but did not measure self-reported treatment adherence (i.e. dosage, frequency, duration, etc) [37]. There are limitations of the Rose questionnaire for assessing angina, including it being short in length, and that subjects with mental health disorders may be more likely to have false positive results [47]. However, large cohort studies showed that the Rose questionnaire had good predictive ability, and false positives from individuals with mental health conditions may be minimal [47–50]. This study did not adjust for the number of visits to a general practitioner in clinics to avoid possible over-adjustment bias. The associations between more comorbidities with better detection of previously undiagnosed NCDs [51], could be mediated by more clinic visits and interactions with healthcare professionals from having more comorbidities [12, 25, 26, 52]. The study was based on 9 NCDs, so future work could examine more conditions. For example, a large-scale Scotland study included 40 NCDs [53]. The study’s cross-sectional design does not allow for causal interpretations, and studies that use prospective cohort designs could examine how comorbidities cause treatment and control of NCDs in subjects that are followed-up prospectively, such as over a few years [6].

### Clinical and policy implications

Our study revealed that subjects with more comorbidity had better detection of NCDs, but control was worse with more comorbidity. There are three principle possible explanations. First, it may be related to poor access to care, whereby patients with multiple chronic conditions are getting treatment for their conditions (perhaps from a pharmacy) but there is a lack of access to care from the primary care system [54–57]. It could also relate to cost, such as a lack of comprehensive universal coverage fee at the point of care [36, 58–60]. Second, it could be from suboptimal adherence to medications [59, 60]. It is well documented that adherence drops as polypharmacy rises [34, 36, 61]. The lack of intentional ongoing monitoring by physicians and allied health professionals might compound this problem, as regular follow-up could include checks on adherence and reinforce the importance of taking the prescribed medications. Third, it could be due to a lack of effectiveness of medication prescribed for each condition in patients with multiple chronic conditions [62]. For most treatments of individual NCDs, the evidence is based on randomised controlled trials which exclude patients with multiple NCDs [62–64]. Thus what works in a patient with a single condition, may not work in a patient with the same NCD with comorbidities [65].

In reality, the problem may be a combination of these three possibilities. Primary healthcare clinicians need to improve the follow up on patients, in order to assess possible discontinuation of certain treatments due to adverse drug events, and the financial constraints that limit patients’ ability to go for follow up check-ups and refilling prescriptions [36, 56, 57]. Another clinical implication is on prioritising NCDs, whereby clinicians should assess NCD profiles of patients, prioritise treatment for patients who would have the greatest clinical benefit of better management and control, and consider personal preferences of patients for managing various NCDs [6, 66–68].

Policies could prioritise NCDs that have higher prevalence or burden, such as focusing on lowering out-of-pocket expenditures for follow-up visits to healthcare providers and medicines [36, 69]. Policies also need to address continual accessibility to healthcare after first diagnosis. In MICs, it is not uncommon for patients to travel from rural residences to the urban areas to seek medical treatment, limiting their ability to regularly visit healthcare services and continue treatment, which results in suboptimal control of NCDs [52, 70, 71]. In addition, there are complexities of insurance policies. Health insurance from employers may be limited to certain districts or urban areas in MICs, and it is possible that healthcare for different conditions are covered by health providers in different physical locations [72, 73]. This problem aggravates the already existing fragmentation of care from utilising multiple health providers and services [53, 74].

Fragmented healthcare is a challenge for patients, policymakers, and clinicians. Reorganising healthcare structures particularly primary care, would benefit individuals with multiple chronic conditions who often have difficulty managing treatment due to polypharmacy, have multiple health providers and medical appointments, high financial burden, and suboptimal control of conditions [6, 11, 53, 74].

## Conclusion

Patients with multiple chronic conditions may have better detection of some chronic conditions, but this does not translate into better management of these conditions. As these patients are high users of health services and are at increased risk of adverse health outcomes, improving their access to care is a priority for health systems. Clinical guidelines should move away from the current focus on single diseases and be tailored to better suit the needs of patients with multiple chronic conditions.

## Data Availability

No additional data available.

## Appendix

**Table 3:** Algorithms used to ascertain presence of non-communicable diseases

NCDs	Self-reported diagnosis	Physical measurement/Symptom-based assessment	Algorithm for physical measurement/symptom-based assessment
Hypertension	Have you ever been diagnosed with high blood pressure?	Take average of three blood pressure readings. High blood pressure is defined as systolic blood pressure≥140mmHg and diastolic blood pressure ≥90mmHg.	High blood pressure is defined as systolic blood pressure≥140mmHg and diastolic blood pressure ≥90mmHg.
Stroke	Have you ever been told by a health professional that you have had a stroke	No symptom-based assessment	No symptom-based assessment
Diabetes	Have you ever been diagnosed with diabetes?	No symptom-based assessment	No symptom-based assessment
Cataract	In the last 5 years, were you diagnosed with a cataract in one or both of your eyes?	No symptom-based assessment	No symptom-based assessment
Arthritis	Have you ever been diagnosed with/told you have arthritis (a disease of the joints, or by other names rheumatism or osteoarthritis)?	Qn 1: During the last 12 months, have you experienced pain, aching, stiffness or swelling in or around the joints (like arms, hands, legs or feet) which were not related to an injury and lasted for more than a month? a. Yes b. No Qn 2: During the last 12 months, have you experienced stiffness in the joint in the morning after getting up from bed, or after a long rest of the joint without movement? a. Yes b. No Qn 3: How long did the stiffness last? a. about 30mins or less b. more than 30 mins Qn 4: Did this stiffness go away after exercise or movement in the joint? a. Yes b. No	Arthritis= Option ‘a’ to both question 1 and question 2. OR Option ‘a’ to both question 3 and question 4.
Angina	Have you ever been diagnosed with angina or angina pectoris?	Qn 1: During the last 12 months, have you experienced any pain or discomfort in your chest when you walk uphill or hurry? a. Yes b. No Qn 2: During the last 12 months, have you experienced any pain or discomfort in your chest when you walk at an ordinary pace on level ground? a. Yes b. No Qn 3: What do you do if you get the pain or discomfort when you are walking? a. stop or slow down b. carry on after taking a pain-relieving medicine that dissolves in your mouth c. carry on walking Qn 4: If you stand still, what happens to the pain or discomfort? a. relieved b. not relieved Qn 5: Will you show me where you usually experience the pain or discomfort? a. (6 & 11), or (7 & 8).	Angina= Option ‘a’ for question 1 OR Option ‘a’ for question 2 OR Option ‘a’ for both question 3 and question 4. OR Option ‘a’ for question 5
Asthma	Have you ever been diagnosed with asthma?	Qn 1: Attacks of wheezing or whistling breathing? a. Yes b. No Qn 2: Attack of wheezing that came on after you stopped exercising or some other physical activity? a. Yes b. No Qn 3: A feeling of tightness in your chest? a. Yes b. No Qn 4: Have you woken up with a feeling of tightness in your chest in the morning or any other time? a. Yes b. No Qn 5: Have you had an attack of shortness of breath that came on without obvious cause when you were not exercising or doing some physical work? a. Yes b. No	Asthma= Option ‘a’ for Qn 1 OR Option ‘a’ for all questions from question 2 to question 5.
Chronic lung disease	Have you ever been diagnosed with chronic lung disease (emphysema, bronchitis, COPD)?	Qn 1: During the last 12 months, have you experienced any shortness of breath at rest? (while awake) a. Yes b. No Qn 2: During the last 12 months, have you experienced any coughing or wheezing for ten minutes or more at a time? a. Yes b. No Qn 3: During the last 12 months, have you experienced any coughing up sputum or phlegm for most days of the month for at least 3 months? a. Yes b. No	Chronic lung disease= Option ‘a’ for Qn 1 OR Option ‘a’ for both question 2 and question 3
Depression	Have you ever been diagnosed with depression?	Qn 1: During the last 12 months, have you had a period lasting several days when you felt sad, empty or depressed? a. Yes b. No Qn 2: During the last 12 months, have you had a period lasting several days when you lost interest in most things you usually enjoy such as personal relationships, work or hobbies/recreation? a. Yes b. No Qn 3: During the last 12 months, have you had a period lasting several days when you have been feeling your energy decreased or that you are tired all the time? a. Yes b. No Qn 4: Was this period [of sadness/loss of interest/low energy] for more than 2 weeks? a. Yes b. No Qn 5: Was this period [of sadness/loss of interest/low energy] most of the day, nearly every day? a. Yes b. No Qn 6: During this period, did you lose your appetite? a. Yes b. No Qn 7: Did you notice any slowing down in your thinking? a. Yes b. No Qn 8: Did you notice any problems falling asleep? a. Yes b. No Qn 9: Did you notice any problems waking up too early? a. Yes b. No Qn 10: During this period, did you have any difficulties concentrating: for example, listening to others, working, watching TV, listening to the radio? a. Yes b. No Qn 11: Did you notice any slowing down in your moving around? a. Yes b. No Qn 12: During this period, did you feel anxious and worried most days? a. Yes b. No Qn 13: During this period, were you so restless or jittery nearly every day that you paced up and down and couldn’t sit still? a. Yes b. No Qn 14: During this period, did you feel negative about yourself or like you had lost confidence? a. Yes b. No Qn 15: Did you frequently feel hopeless- that there was no way to improve things? a. Yes b. No Qn 16: During this period, did your interest in sex decrease? a. Yes b. No Qn 17: Did you think of death, or wish you were dead? a. Yes b. No Qn 18: During this period, did you ever try to end your life? a. Yes b. No	Group A: Score=1 if option ‘a’ to question 1 Score=1 if option ‘a’ question 2 Score=1 if option ‘a’ to question 3 Addition of score= score for Group A Group B: Score=1 if option ‘a’ to either question 8 or question 9 Score=1 if option ‘a’ to either question 7 or question 10 Score=1 if option ‘a’ to either question 11 or question 13 Score=1 if option ‘a’ to either question 14 or question 15 Score=1 if option ‘a’ to either question 17 or question 18 Addition of score= score for Group B Group C: Score=1 if option ‘a’ to question 6 Group D: Score=1 if option ‘a’ to question 12 Group E: Score for Group B + Score for Group C + Score for Group D Group F: Score=1 if option ‘a’ to question 4 Group G: If Score for Group A ≥2, AND Score for Group F is =1, add the score for Score for Group A + Score for Group E Depression= Score for Group G ≥4

**Table 4:** Definitions of prevalence of each chronic condition being undiagnosed, untreated, and uncontrolled

Non- communicable disease	Prevalence				
	Undiagnosed^a^	Untreated		Uncontrolled	
		unT-last 2 weeks^b^ (untreated in the last 2 weeks)	unT-last 12 months^b^ (untreated in last 12 months)	unC-T-last 2 weeks^b^ (treated in the last 2 weeks, and uncontrolled)	unC-T-last 12 months^b^ (treated in the last 12 months, and uncontrolled)
Hypertension	All subjects who self- reported not being diagnosed with hypertension, but have hypertension based on symptom-based assessment.	All subjects who self-reported being diagnosed with hypertension, but self-reported not taking treatment in the last 2 weeks. Treatment that they self-reported taking include medications or other treatment such as a weight loss program or changing eating habits.	All subjects who self-reported being diagnosed with hypertension, but self-reported not taking treatment in the last 12 months. Treatment that they self-reported taking include medications or other treatment such as a weight loss program or changing eating habits.	All subjects who are self-reported taking treatment for hypertension in the last 2 weeks, but have hypertension based on BP measurement.	All subjects who are self-reported taking treatment for hypertension in the last 12 months, but have hypertension based on BP measurement
Angina	All subjects who self- reported not being diagnosed with angina, but have angina based on symptom-based assessment.	All subjects who self-reported being diagnosed with angina, but self-reported not taking treated in the last 2 weeks. Treatment that they self-reported taking include medications or other treatment.	All subjects who self-reported being diagnosed with angina, but self-reported not taking treatment in the last 12 months. Treatment that they self-reported taking include medications or other treatment.	All subjects who self-reported taking treatment for angina in the last 2 weeks, but have angina based on symptom-based assessment.	All subjects who self-reported taking treatment for angina in the last 12 months, but have angina based on symptom-based assessment.
Arthritis	All subjects who self- reported not being diagnosed with arthritis, but have arthritis based on symptom-based assessment.	All subjects who self-reported being diagnosed with arthritis, but self-reported not taking treatment in the last 2 weeks. Treatment that they self-reported taking include medications or other treatment.	All subjects who self-reported being diagnosed with arthritis, but self-reported not taking treatment in the last 12 months. Treatment that they self-reported taking include medications or other treatment.	All subjects who self-reported taking treatment for arthritis in the last 2 weeks, but have arthritis based on symptom-based assessment.	All subjects who self-reported taking treatment for arthritis in the last 12 months, but have arthritis based on symptom-based assessment.
Asthma	All subjects who self- reported not being diagnosed with asthma, but have asthma based on symptom-based assessment.	All subjects who self-reported being diagnosed with asthma, but self-reported not taking treatment in the last 2 weeks. Treatment that they self-reported taking include medications or other treatment.	All subjects who self-reported being diagnosed with asthma, but self-reported not taking treatment in the last 12 months. Treatment that they self-reported taking include medications or other treatment.	All subjects who self-reported taking treatment for asthma in the last 2 weeks, but have asthma based on symptom-based assessment.	All subjects who self-reported taking treatment for asthma in the last 12 months, but have asthma based on symptom-based assessment.
Chronic Lung Disease	All subjects who self- reported not being diagnosed with chronic lung disease, but have chronic lung disease based on symptom- based assessment.	All subjects who self-reported being diagnosed with chronic lung disease, but self-reported not taking treatment in the last 2 weeks. Treatment that they self-reported taking include medications or other treatment (like oxygen).	All subjects who self-reported being diagnosed with chronic lung disease, but self-reported not taking treatment in the last 12 months. Treatment that they self-reported taking include medications or other treatment (like oxygen).	All subjects who self-reported taking treatment for chronic lung disease in the last 2 weeks, but have chronic lung disease based on symptom-based assessment.	All subjects who self-reported taking treatment for chronic lung disease in the last 12 months, but have chronic lung disease based on symptom-based assessment.
Diabetes	Not applicable (Diabetes has no symptom-based assessment)	All subjects who self-reported being diagnosed with diabetes, but self-reported not taking treatment in the last 2 weeks. Treatment that they self-reported taking include insulin or other blood sugar lowering medications, special diet, exercise regime, or weight control program, as recommended by a health professional.	All subjects who self-reported being diagnosed with diabetes, but self-reported not taking treatment in the last 12 months. Treatment that they self-reported taking include insulin or other blood sugar lowering medications only.	Not applicable (Diabetes has no symptom-based assessment)	Not applicable (Diabetes has no symptom-based assessment)
Cataract	Not applicable (Cataract has no symptom-based assessment)	Not applicable (Subjects self-reported not being treated in the last 5 years, not in the last 2 weeks)	All subjects who self-reported being diagnosed with cataract, but self-reported not taking treatment in the last 5 years. Treatment refers to eye surgery.	Not applicable (Cataract has no symptom-based assessment)	Not applicable (Cataract has no symptom-based assessment)
Depression	All subjects who self- reported not being diagnosed with depression, but have depression based on symptom-based assessment.	All subjects who self-reported being diagnosed with depression, but self-reported not taking treatment in the last 2 weeks. Treatment that they self-reported taking include medications or other treatment. Other treatment can include attending therapy or counselling sessions.	All subjects who self-reported being diagnosed with depression, but self-reported not taking treatment in the last 12 months. Treatment that they self-reported taking include medications or other treatment. Other treatment can include attending therapy or counselling sessions.	All subjects who self-reported taking treatment for depression in the last 2 weeks, but has depression based on symptom-based assessment.	All subjects who self-reported taking treatment for depression in the last 12 months, but has depression based on symptom-based assessment.
Stroke	Not applicable (Stroke has no symptom-based assessment)	All subjects who self-reported being diagnosed with stroke, but self-reported not taking treatment in the last 2 weeks. Treatment that they self-reported taking include to medications or other treatment.	All subjects who self-reported being diagnosed with stroke, but self-reported not taking treatment in the last 12 months. Treatment that they self-reported taking include medications or other treatment.	Not applicable (Stroke has no symptom-based assessment)	Not applicable (Stroke has no symptom-based assessment)

^a^Regarding self-reporting diagnoses, we defined respondents as self-reporting a non-communicable disease if they answered affirmatively to: “Have you ever been diagnosed with…?”

^b^All subjects who self-reported taking medication or other treatment are a perfect subset of those who self-reported being diagnosed, because the questionnaire only asks subjects if they are taking treatment, if they self-report affirmatively to being diagnosed

**Table 5:** Prevalence of comorbidity for each non-communicable disease

		Non-communicable disease								
		Hypertension	Angina	Arthritis	Asthma	Chronic Lung Disease	Diabetes	Cataract	Depression	Stroke
Prevalence of subjects (%)	No comorbidity	38.77	19.59	44.80	34.11	34.87	29.25	32.39	39.68	22.48
	1 comorbidity	29.42	30.54	25.91	24.93	27.98	23.52	25.71	24.45	30.06
	2 comorbidities	17.21	24.54	15.83	15.70	12.46	18.87	20.14	20.79	21.04
	3 comorbidities	7.48	14.10	6.37	12.36	12.46	15.27	13.33	5.93	12.16
	4+ comorbidities	7.11	11.23	7.10	12.89	12.23	13.10	8.43	9.15	14.26

**Table 6:** Prevalence of undiagnosed subjects for each non-communicable disease, with increasing number of comorbidities

		Undiagnosed non-communicable disease					
		Hypertension	Angina	Arthritis	Asthma	Chronic Lung Disease	Depression
Prevalence of undiagnosed subjects (%)	No comorbidy	72.25	78.55	48.67	55.51	50.82	71.52
	1 comorbidity	46.34	60.82	41.76	56.76	50.92	69.04
	2 comorbidities	30.61	44.95	33.04	54.28	55.44	54.98
	3 comorbidities	20.53	32.84	24.48	46.58	48.29	67.12
	4+ comorbidities	16.61	28.63	21.77	64.09	36.38	63.45

**Table 7:** Prevalence of undiagnosed subjects for each non-communicable disease (NCD), with diagnosed concordant NCDs only, discordant NCDs only, and depression only

		Undiagnosed non-communicable disease					
		Hypertension	Angina	Arthritis	Asthma	Chronic Lung Disease	Depression
Prevalence of undiagnosed subjects (%)	Diagnosed with only concordant NCDs	26.83	39.20	NA	50.75	42.34	NA
	Diagnosed with only discordant NCDs	34.44	48.74	35.63	52.05	48.16	64.92
	Diagnosed with only depression	35.49	65.45	34.45	47.70	57.51	NA

**Table 8:** Prevalence of untreated subjects for each non-communicable disease, with increasing comorbidities. (untreated in the last 2 weeks)

		Untreated non-communicable disease (in the last 2 weeks)							
		Hypertension	Angina	Arthritis	Asthma	Chronic Lung Disease	Diabetes	Depression	Stroke
Prevalence of untreated subjects (%)	No comorbidity	42.93	56.15	62.16	51.80	92.96	30.64	69.11	85.39
	1 comorbidity	35.72	49.76	62.20	54.76	84.84	32.85	67.67	55.63
	2 comorbidities	25.95	38.20	51.51	23.28	87.60	37.28	72.29	42.43
	3 comorbidities	20.03	30.81	48.93	36.61	94.57	22.65	78.51	42.16
	4+ comorbidities	25.08	18.97	44.50	51.68	96.37	50.40	74.33	54.05

**Table 9:** Prevalence of untreated subjects for each non-communicable disease (NCD), for comorbidity with only concordant NCDs, only discordant NCDs, and only depression. (untreated in the last 2 weeks)

		Untreated non-communicable disease (NCD) (in the last 2 weeks)							
		Hypertension	Angina	Arthritis	Asthma	Chronic Lung Disease	Diabetes	Depression	Stroke
Prevalence of untreated subjects (%)	Comorbidity with only concordant NCDs	27.39	40.49	NA	59.47	89.09	31.46 (concordant with hypertension, angina, and/or stroke); 25.85 (concordant with cataract)	NA	46.50
	Comorbidity with only discordant NCDs	38.51	59.21	55.34	40.85	89.76	37.04	71.34	67.84
	Comorbidity with only depression	35.81	30.41	70.33	89.11	98.16	0.40	NA	88.85

**Table 10:** Prevalence of untreated subjects for each non-communicable disease, with increasing comorbidity. (untreated in the last 12 months)

		Untreated non-communicable disease (NCD) (in the last 12 months)								
		Hypertension	Angina	Arthritis	Asthma	Chronic Lung Disease	Diabetes	Cataract (last 5 yrs)	Depression	Stroke
Prevalence of untreated subjects (%)	Comorbidity with only concordant NCDs	8.72	12.48	NA	43.09	79.17	19.64 (concordant with hypertension, angina, and/or stroke); 23.17 (concordant with cataract)	61.96	NA	34.78
	Comorbidity with only discordant NCDs	19.16	30.68	28.96	28.98	84.70	24.81	65.05	63.81	63.21
	Comorbidity with only depression	22.76	17.10	33.84	4.11	98.16	65.83	92.27	NA	92.66

**Table 11:** Prevalence of untreated subjects for each non-communicable disease (NCD), for comorbidity with only concordant NCDs, only discordant NCDs, and only depression. (untreated in the last 12 months)

		Untreated non-communicable disease (NCD) (in the last 12 months)								
		Hypertension	Angina	Arthritis	Asthma	Chronic Lung Disease	Diabetes	Cataract	Depression	Stroke
Prevalence of untreated subjects (%)	0 comorbidity	25.02	33.34	35.15	20.07	84.14	24.82	55.71	63.12	81.46
	1 comorbidity	16.19	18.49	31.88	33.20	78.11	21.84	62.71	72.19	48.85
	2 comorbidities	10.94	14.45	28.91	20.02	82.69	29.41	62.43	59.68	28.21
	3 comorbidities	8.67	11.64	28.02	29.22	90.08	13.12	50.26	52.32	8.14
	4+ comorbidities	6.28	6.75	19.31	22..39	94.34	34.80	77.69	58.37	17.85

**Table 12:** Prevalence of uncontrolled subjects for each non-communicable disease, with increasing comorbidity. (treated in the last 2 weeks)

		Uncontrolled non-communicable disease (NCD) (treated in the last 2 weeks)					
		Hypertension	Angina	Arthritis	Asthma	Chronic Lung Disease	Depression
Prevalence of uncontrolled subjects (%)	0 comorbidity	65.41	78.87	70.99	82.22	74.00	68.94
	1 comorbidity	70.45	76.22	71.85	86.14	92.13	14.40
	2 comorbidities	76.95	83.27	83.68	60.91	80.63	49.12
	3 comorbidities	75.60	89.95	90.27	95.53	93.60	44.55
	4+ comorbidities	78.46	94.32	95.99	63.17	50.84	79.57

**Table 13:** Prevalence of uncontrolled subjects for each non-communicable disease (NCD), for comorbidity with only concordant NCDs, only discordant NCDs, and only depression. (treated in the last 2 weeks)

		Uncontrolled non-communicable disease (NCD) (treated in the last 2 weeks)					
		Hypertension	Angina	Arthritis	Asthma	Chronic Lung Disease	Depression
Prevalence of uncontrolled subjects (%)	Comorbidity with only concordant NCDs	73.49	80.28	NA	84.71	99.99	NA
	Comorbidity with only discordant NCDs	70.43	75.87	81.81	66.33	81.08	36.99
	Comorbidity with only depression	60.62	86.99	82.31	99.96	99.99	NA

**Table 14:** Prevalence of uncontrolled subjects for each non-communicable disease, with increasing comorbidity (treated in last 12 months)

		Uncontrolled non-communicable disease (NCD) (treated in the last 12 months)					
		Hypertension	Angina	Arthritis	Asthma	Chronic Lung Disease	Depression
Prevalence of uncontrolled subjects (%)	0 comorbidity	63.43	73.09	66.56	78.30	60.50	68.83
	1 comorbidity	64.21	72.46	69.86	73.17	89.96	40.21
	2 comorbidities	74.70	77.12	83.94	65.36	54.62	66.71
	3 comorbidities	72.32	83.29	85.92	93.47	82.47	46.76
	4+ comorbidities	67.49	90.97	81.42	86.88	52.97	77.41

**Table 15:** Prevalence of uncontrolled subjects for each non-communicable disease (NCD), for comorbidity with only concordant NCDs, discordant NCDs, and depression. (treated in the last 12 months)

		Uncontrolled non-communicable disease (NCD) (in last 12 months)					
		Hypertension	Angina	Arthritis	Asthma	Chronic Lung Disease	Depression
Prevalence of uncontrolled subjects (%)	Comorbidity with only concordant NCDs	63.43	84.36	NA	86.93	84.48	NA
	Comorbidity with only discordant NCDs	67.35	63.19	77.46	65.13	72.96	57.77
	Comorbidity with only depression	52.27	88.30	63.19	62.69	99.99	NA

## Abbreviations

AOR: Adjusted odds ratio
CLD: Chronic lung disease
MICs: Middle-income countries
NCD: Non-communicable disease;
WHO SAGE: World Health Organisation Study of Global Ageing and Adult Health
